# Protein Source and Intake Effects on Diet Digestibility and N Excretion in Horses—A Risk of Environmental N Load of Horses

**DOI:** 10.3390/ani11123568

**Published:** 2021-12-15

**Authors:** Markku Saastamoinen, Susanna Särkijärvi, Heli Suomala

**Affiliations:** 1 Production Systems, Natural Resources Institute Finland (Luke), 31600 Jokioinen, Finland; susanna.sarkijarvi@roviopetfoods.fi; 2 Department of Animal Science, University of Helsinki, 00790 Helsinki, Finland; heli.suomala@hevostietokeskus.fi

**Keywords:** horse, nutrition, feeding, feeds, environment

## Abstract

**Simple Summary:**

The aim of this experiment was to study how protein source and intake effect diet digestibility and N excretion in adult horses. The results showed that protein feeds improved diet DM, OM and CP digestibilities compared to forage diets. Urine excretion was greater for forage-only diets compared with diets including protein supplements. Horses excreted more nitrogen in their urine (85–139 g/d) than in dung (42–52 g/d), and N excretion differed between the diets. Horses on a haylage-only diet excreted 51.6 g N in faeces/day and on hay-only diet 51.4 g N/day. On the other hand, when protein content in forages increased, N excretion via urine increased. Horses excreted less N in urine with hay-only diet (64 g/d) than with haylage-only (104 g/d) or protein-supplemented diets (105–111 g/d). The results indicated that feed choices affected the amount of nitrogen excreted. Feeding recommendations should consider not only the horse category and work level, but also the protein source. When good quality protein is fed, smaller N intakes can be applied to reduce the N excretion via urine and dung. At the farm level, improved understanding of feed quality, as well as feeding planning and practices, is a way to decrease the risk of N leaching and evaporation.

**Abstract:**

Six Finnhorse mares were used in a digestibility trial, in which six typical horse diets were compared. The diets were: (A) haylage 100%; (B) hay 100%; (C) hay 70% and oats 30%; (D) hay 70% and soybean meal + oats 30%; (E) hay 70%, rapeseed meal + oats 30% and (F) hay 70 %, linseed meal + oats 30%. The trial was conducted according to an unbalanced 6 × 4 Latin square design with four 3-week experimental periods. The experimental period consisted of 17-day preliminary feeding which was followed by a 4-day total faecal and urine collection periods to evaluate N excretion. The digestibilities of DM (*p* < 0.001) and OM (*p* < 0.001) in the haylage-only diet were lower compared to the other diets. The supplemental protein feeds improved the diet digestibility of CP (*p* = 0.002) compared to a hay + oats diet. Furthermore, the DM (*p* = 0.019), OM (*p* = 0.006), and CP (*p* = 0.016) digestibilities of the soya-supplemented diet were better than those of the rapeseed- and linseed-supplemented diets. Faecal excretion was greater for haylage (19.3 kg fresh faeces and 3.6 kg DM/day) and hay (18.7 kg fresh faeces and 3.6 kg DM/day) diets (*p* < 0.001) compared with the other diets. Urine excretion was also greater for forage-only diets compared with diets including protein supplements. Horses excreted 14.0 L urine/day on haylage-only diet (*p* = 0.026) and 14.3 L/day on a hay-only diet (*p* = 0.003). Horses excreted more nitrogen in their urine than in dung. N excretion differed between the diets. Horses on a haylage-only diet excreted 51.6 g N in faeces/day and on hay-only diet 51.4 g N/day. On the other hand, when protein content in forages increased, N excretion via urine increased (haylage vs. dried hay). Horses excreted less N in urine with hay-only diet than with haylage-only or protein-supplemented diets (*p* < 0.001). When N excreted in faeces and urine was counted together, horses excreted less N with a hay-only diet (*p* < 0.001) than with a supplemented one (oats and/or protein feeds). The results showed that feed choices affected the amount of nitrogen excreted. Feeding recommendations should consider not only the horse category and work level, but also the protein source. When good quality protein is fed, smaller N intakes can be applied to reduce the N excretion via urine and dung. At the farm level, improved understanding of feed quality, as well as feeding planning and practices, is a way to decrease the risk of N leaching and evaporation.

## 1. Introduction

Horses require various amounts of protein (nitrogen). Feeds fed to horses contain different quantities of protein, which is formed of amino acids (AA) [1]. Nitrogen content in herbage ranges typically from 10 to 50 g/kg DM [2]. Plants also contain non-protein nitrogen (NPN), which consists of soluble and non-soluble fractions. The proportion of NPN of total N in plants is 15–20% [1]. It is not usable for horses but may support microbial populations of the digestive tract [1].

Protein requirements are increased especially in growing horses, lactating mares and horses in training. Instead, the protein needs of sedentary horses are not high. When the requirements are high, the protein content is difficult or even impossible to fulfil with basic feeds (e.g., preserved forages) of medium or low quality [3,4,5], and diets are supplemented, or are recommended to be supplemented, with protein-rich feeds. In addition to the CP content and its availability, the protein quality, i.e., amino the acid composition of the feed, is important. The better the AA profile in feeds corresponds to the needs of the horse, the better the horse can utilize the protein, and the less protein (nitrogen) can be fed.

There are two typical conflicts for protein use in horse nutrition. Sedentary horses and those in very light work have low protein needs and should have low CP intakes, but their diet CP content may often be too high. On the other hand, quite high intakes are required by athletic horses, growing horses and lactating mares, but their requirements are not met [1]. Recent studies show that it is common in practice to feed horses an excess of nutrient requirements, including protein (e.g., [6,7]). Forage-only diets covering energy requirements in trained horses usually contain excess protein compared to needs [8,9]. The horse excretes most of the nitrogen as urea in the urine, some of the nitrogen is excreted in the dung. Factors affecting the horse, excess protein may cause increased water consumption, sweating and urine excretion, disturbing the horse’s fluid balance and possible stressing the metabolism when excess unabsorbed protein (nitrogen) forms urea in the liver (e.g., [3,8]). The horse’s daily urine output is 3 to 18 mL/kg body weight (e.g., [10]). Optimizing CP intake is important, because a high CP intake also increases renal energy loss [11] when N from excess CP intake is excreted as urea.

Excreted nitrogen as a consequence of excess nitrogen intake may increase the N load on the environment [12]. In the stable, urine is absorbed into the litter, but in pasture feeding and plant-free paddocks, it gets into the soil and water bodies. In paddocks and pastures there may be particular hot spots, i.e., defecation and urination areas and feeding places [13,14,15]. Nitrogen in manure that has not been stored for protection from rain and runoff, can also leach into the environment. The N leached from dung was mainly in organic form [16].

N is important nutrient of plants [17]. Urea-N is degraded in the soil by the enzyme urease to ammonia (NH_3_^−^) and carbon dioxide (CO_2_). Further, ammonia is oxidized in the soil to ammonium nitrogen (NH_4_^+^). In terrestrial nitrification, certain microbes oxidize ammonium nitrogen (NH_4_^+^) to nitrite (NO_2_^−^) and further to nitrate nitrogen (NO_3_^−^), which can leach with heavy rainfall or melting waters. A by-product of the nitrification produces hydrogen which acidifies the earth (free hydrogen ion H^+^). In a condensed soil with anaerobic conditions, denitrification, i.e., the reduction of nitrate as a result of the action of anaerobic bacteria to gaseous nitrogen compounds to nitrous oxide (N_2_O) or nitrogen gas (N_2_), may occur, nitrous oxide (NO_2_) being an ozone-depleting greenhouse gas [17]. Therefore, in addition to N leaching to waters, N evaporates in the form of NH_3_, N_2_ and NO gases [18,19]. Ammonia volatilization is generally the major pathway for N losses from manure [20,21]. In Europe [22], and according to United States Environmental Protection Agency USEPA [12] also in USA, the main source of ammonia emissions is agriculture, including animal husbandry.

The number of horses has increased in many countries, with horses increasingly used for hobbies and sports, as well as in recreation and therapy (horse-assisted therapy) [23]. Few studies have examined horses’ N excretion, although horse husbandry causes both runoffs and gas emissions. Because paddocks and manure are an important source of N leaching and emissions, it is important to optimize the nutrition and especially protein intake of horses. This also has been economic impact, because protein is a costly component of nutrition. The hypothesis was that the diet and protein source influence the amount of nitrogen excreted by horses in their dung and urine. This experiment was the second part of the study dealing with possible harmful environmental impacts of horse husbandry (*p*- and N-leaching).

## 2. Materials and Methods

A digestibility trial was conducted with six forage-based diets typically fed to horses in Finland. The study was conducted at the facilities of the Natural Resources Institute Finland (Luke) in Southwest Finland. In animal handling and sample collection, the European Union recommendation directives (2010/63/EU) and national animal welfare and ethical legislation set by the Ministry of Agriculture and Forestry of Finland were followed carefully. The experimental procedures were evaluated and approved by the National Ethical Committee for animal experiments (https://www.avi.fi/web/avi/elainkoelautakunta-ella, accessed on: 15 January 2021) (ESAVI/8331/04.10.07/2013).

### 2.1. Horses and Their Management

Six adult Finnhorse mares (6–15-years-old; initial BW 565 ± 30.0 kg, mean BCS 6 = moderately fleshy [24]) owned by Luke were used in the study. All the experimental horses had the same managing and feeding history before the trial. They were de-wormed with ivermectin (Equimax^®^, Virbac, France) before the experiment. Dental care and vaccinations had been carried out regularly prior to the experiment. The horses were individually housed in stalls (3 m × 3 m) with peat as bedding. The horses were ridden daily for one hour (light work: walk, trot) and freely exercised in groups in outdoor paddocks (with sand grounds) for 2–4 h, except during the collection period, when they were led in a walk by a rope in the stable corridors (consisting of two connected 32-m corridors with concrete and asphalt floors) for 15 min. In the paddocks, the horses had muzzle to prevent sand eating.

The study method was a total collection of faeces. The experimental design was arranged as unbalanced 6 × 4 Latin Squares. This experiment was part of a series of two studies examining environmental impacts of horse husbandry—the first part has been already published [25]. Similar study design was applied in both studies. The experiment consisted of six treatments and four 21-day periods. Each period started with a five-day feed change period followed by 12 days of adaptation to the new diet, and a four-day period of collecting faeces and urine samples.

The BW (electronic animal scale Lahden Vaaka/Lahti Precision Ltd., Lahti, Finland) and BCS [24] of the horses was monitored after each collection period to control possible changes and adjust the individual energy intakes if necessary.

### 2.2. Experimental Feeds and Feeding

The horses were individually fed at a level of 65–75 g DM kg^−1^W^0.75^, corresponding to the feeding level recommended in light work in accordance with the Finnish Feed Tables and Feeding Recommendations [26]. The diets were formulated and adjusted to be as isocaloric and isonitrogenous as possible. The horses were fed three times per day (at 6 a.m., 12 noon and 6 p.m.), except in the mornings of blood sampling days, when the forages were fed at 7.30 and concentrates at 8 p.m. Forages were fed before the administration of the concentrates.

Two of the diets were roughage-only diets. The four other diets consisted of dried hay and rolled (crimped) oats, supplemented by a protein source (soya groats, rapeseed groats, linseed groats; Suomen Rehu Ltd., Seinäjoki, Finland) commonly fed to horses in Finland, to meet the protein requirements. The forage-to-concentrate ratio in these diets was 70:30. In addition, horses were supplemented with a mineral mixture (100 g/d, Hiven Ltd.), and salt blocks (100% NaCl, Milka, Biofarm Ltd., Karkkila, Suomi) were available to all the horses.

The diets were (dry matter basis) (A) haylage 100%; (B) dried hay 100%; (C) dried hay 70% + rolled oats 30%; (D) dried hay 70% + soya groats + rolled oats 30%; (E) dried hay 70% + rapeseed groats + rolled oats 30%; and (F) dried hay 70% + linseed groats + rolled oats 30%. Linseed groats were soaked for 15 min before feeding. All other feeds were fed dry. The average diet formulations (kg/d, as fed) are presented in Table 1.

Dried hay was selected for basic forage, because it is the major forage fed to horses in Finland, and its nutritional variation is smaller than in haylages [4]. The protein supplements were used to compare imported soya with domestic protein sources (linseed, rapeseed by products), which have been proved to be suitable feeds in horse nutrition [27,28].

The dried hay was produced by a local farmer in Ypäjä (60°48.34 N, 23°16.35 E). The haylage (Prohay Ltd., Punkalaidun, Finland, 61°06.40 N, 23°06.20 E) was packed in 20 kg airtight plastic packages and purchased from the producer. The oats were produced by Luke.

### 2.3. Feed Sampling and Analyses

Feed samples and possible refusal feeds were collected daily and stored at –20 °C until analysis. The samples were analysed at the Luke Laboratories (Jokioinen, Finland) for dry matter (DM), crude protein (CP), as well as NDF and ADF. Nitrogen (N) was analysed with the Khjeldal method (AOAC-984.13), using a Foss Khjeltec 2400 analyzer (Foss Analytical AB, Höganäs, Sweden), and the CP content was calculated as 6.25 × N. Ash was determined according to the method of AOAC-942.05 by firing the sample at 600 °C for 2 h or at 510 °C for 16 h.

NDF and ADF were analysed using an ANKOM 220 Fiber Analyzer, according to instructions by the manufacturer (ANKOM Technology, Macedon NY 14502, USA: aNDF Method (Method 6) and ADF Method (Method 5), Filter Bag Technique for A200 and A200I, using 25 µm nylon bags). Detergent solution was made according to Robertson et al. [29]. NDF is expressed without containing residual ash. The chemical composition of the feeds is presented in Table 2.

### 2.4. Faeces and Urine Sampling and Analyses

For the time of the collection period, peat bedding was removed and changed to rubber mats. The salt blocks were also removed to avoid increased water intake and its resulting urine excretion.

Daily excreted faeces was collected from the rubber mats, weighed and put into buckets with a lid. The faeces was mixed carefully, and samples of 12% of the total weight were taken (stored at −7.5 °C) for laboratory analyses.

Daily excreted urine from each horse was collected in a plastic bottle using urine collecting harnesses (Equine Diaper^®^, Equisan Marketing PTY Ltd., Southbank, Australia) and weighted. The urine was mixed thoroughly, and a 30 mL sample was taken immediately after collection with a plastic syringe. Urine samples from each horse obtained during the collection period were pooled and stored at −34 °C in a plastic (250 mL) bottle. The remainder of the urine was sieved into a covered 10-L plastic bucket containing 200 mL of 5-M sulphuric acid to prevent nitrogen from evaporating from the urine, and to identify if there were essential differences in the analysed N content due to the method. The pH of acidified urine was monitored with pH papers, and acid was added as needed. A total of 100 mL of acidified urine sample per horse was taken twice a day, and the samples were combined in one-litre plastic bottles and stored at −7.5 °C. All contaminated urine (e.g., with dung) were defined as waste. The amount of waste faeces was weighed but not utilized in the analysis.

On some days, the collection of all the urine was unsuccessful, with losses due to difficulties in keeping and fitting the harnesses tightly on the mares. The daily amount of urine was therefore calculated based on its creatinine concentration in the urine [30]: Y = 24.3 + (14067/x), where y is the urine mount, and x is the creatinine concentration in the urine (creatinine molecular weight 0.13113 g/mmol). The N balances were not calculated because of the uncertain urine collection method and calculated values.

The faeces and urine samples were analysed at Luke Laboratories. Faeces samples were analysed using the same methods for dry matter (DM), crude protein (CP), NDF, and ADF and ash as feed samples, as described above. The faeces were dried in an oven for 20 h (2 h at + 60 °C, 18 h at +110 °C) and milled (1 mm sieve) for the analyses. The fresh sample was also analysed for N content using the Khjeldal method. Urine samples were analysed for nitrogen (Khjeldal method) and creatinine concentrations for both the pure and acid-treated urine. The analysis of creatinine was based on the photometric method of Jaffe (see, e.g., [31]), using a wavelength of 510 nm. The mean urinary specific gravity of the horses reported in the literature was 1.034 g/L [32,33,34,35,36].

### 2.5. Blood Sampling and Analyses

Blood samples were collected 90 min after the morning meal on the third day of the collection period. A blood sample was drawn from the jugular vein into two 10 mL sample tubes (BD Vacutainer K2E 18.0 mg REF 367525, BD Plymouth, UK). The samples were analysed for leukocytes, erythrocytes, haemoglobin and haematocrit to follow the health status of the horses during the study period. Serum urea and total protein were analysed to describe the protein balance of the horses. The analyses were performed in a clinical authorized laboratory (Ellab Ltd., Ypäjä, Finland) using clinical chemistry methods.

### 2.6. Statistical Analyses

Differences in digestibility and excretion parameters between the diets were statistically analysed using the SAS (SAS 9.4, 2014) Mixed procedure (SAS Institute, Cary, NC, USA) applying the following statistical model: Y_ijk_ = µ_ijk_ + a_i_ + p_j_ + d_k_ + e_ijk_, where µ_ijk_ is the overall mean, a_i_ is the random effect of the animal (i = 1…6), p_j_ is the fixed effect of the period (j = 1…4), d_k_ is the fixed effect of the diet (k = 1…6) and e_ijk_ is the normally distributed error, with a mean of 0 and variance δ^2^. Statistical reliability was confirmed according to the Shapiro–Wilk-test (*p* > 0.05) and the normal distribution of residuals. The differences between the diets were tested with orthogonal contrasts: (1) A vs. B and C–F; (2) B vs. C–F; (3) C vs. D, E and F; (4) D vs. E and F and (5) E vs. F. Concerning the protein supplements, contrasts were selected to compare the diets containing domestic feedstuffs (linseed, rapeseed by products) with imported protein feed (soya).

## 3. Results

The horses were clinically healthy during the study. They maintained their BCS and body weight.

### 3.1. Feed and Nutrient Intakes

The feeds fed had good palatability for all the horses. Few, if any, leftovers were collected.

Collecting the feed samples during the course of the study showed that the DM content of the haylage was lower in the first period than in that obtained for the other periods (59.9 vs. 62.3–65.1%). This caused an essentially smaller DM intake of the horse ingested haylage, resulting in a smaller CP intake. This abnormal CP intake was therefore excluded from the statistical analyses in the fourth period (Table 3). Another horse had an exceptionally negative ash digestibility (−0.00326) and may have eaten sand (in spite of its muzzle), resulting in higher ash excretion in the faeces than ingested in the feed. This value was also excluded from the statistical analysis. These exclusions resulted in 23 observations for CP intake (Table 3) as well as digestibility and excretion of Ash, instead of 24 observations for the other parameters (Table 4 and Table 5).

The diets were not isonitrogenous. The lowest CP contents, and thus, intakes were obtained for the hay-only diet (B); the mean ingested CP and dCP amounts were 0.67 kg/d and 0.42 kg/d, respectively. These were statistically significantly lower (*p* < 0.001 for CP, *p* = 0.007 for dCP) than the intakes in supplemented diets (C, D, E, F) (0.72–0.89 kg CP, 0.45–0.58 dCP). The DM and OM intakes were smallest in horses in a haylage-only diet (Diet A) (*p* < 0.001). Horses fed with 100% dried hay (Diet B) ingested more fibre (NDF, ADF) than horses in supplemented diets (*p* < 0.001).

Diets supplemented with protein feeds (soya, rapeseed, linseed) offered naturally more CP (*p* < 0.001) and dCP (*p* = 0.008) than the hay diet supplemented only with oats (C). Horses supplemented with rape seed (Diet E) ingested somewhat more CP than those supplemented with flax seed (F) (*p* = 0.026).

Energy intake (ME MJ/day) was smallest in the haylage-only diet, and in the hay-only diet, it was smaller than in the supplemented diets. The differences of ME intakes were not tested statistically.

### 3.2. Diet Digestibility

The digestibilities of DM (*p* < 0.001) and OM (*p* < 0.001) in the haylage-only diet were lower than in the other experimental diets (Table 4). The horses fed with a hay-only diet (B) digested the fibre components (NDF *p* = 0.051; ADF *p* = 0.008) better than the horses in the supplemented diets (C–F). Instead, the supplemental protein feeds (D, E, F) improved the diet digestibility of CP (*p* = 0.002) compared to the hay + oats diet (B).

Furthermore, the DM (*p* = 0.019), OM (*p* = 0.006 and CP (*p* = 0.016) digestibilities of the soya-supplemented diet (D) were better than those of the rapeseed- (E) and linseed- (F) supplemented diets. There were no statistically significant differences in the digestiblities between the rapeseed- and linseed-supplemented feedings.

### 3.3. Excretion of Faeces, Urine, Nutrients and Nitrogen

#### 3.3.1. Faeces

Horses on the haylage-only diet excreted larger amounts of fresh faeces (19.3 kg/d) than horses on the other diets (*p* < 0.001) (Table 5). The hay-only diet resulted in a larger daily faeces amount compared to the supplemented diets (*p* < 0.001). In addition, diet D supplemented with soya groats produced more faeces DM than the other protein feed supplemented diets (E and F) (*p* = 0.012). Faeces DM was largest (3.6 kg/d) for horses on haylage- and hay-only diets, and smallest on the hay + oats diet (3.1 kg/d) (*p* < 0.001). Horses on the hay-only diet produced more both fresh dung and faeces DM (*p* < 0.001) than horses fed with supplemented diets.

#### 3.3.2. Urine

Urine excretion was largest (and almost equal) for the forage-only diets (difference not tested), being 14.0 and 14.3 L per day for the haylage-only (A) and hay-only (B) diets respectively. For the haylage-only diet, the excretion of urine was statistically significantly (*p* = 0.026) larger than in all the other diets. The difference was also significant between the hay-only diet and the supplemented diets (*p* = 0.003).

#### 3.3.3. Nutrients

Horses on haylage- and hay-only diets excreted more DM and OM in their faeces than those on supplemented diets (*p* < 0.001). The forage-only diets also produced statistically significantly (*p* = 0.002–0.001) larger excretion of CP and the fibre fractions than the supplemented diets (Table 6). Horses fed with the hay-only diet excreted all nutrients (except ash) more than the horses on the other diets (*p* < 0.001). The soya-supplemented diet produced less dung DM (*p* = 0.012), OM (*p* = 0.004), CP (*p* = 0.007) as well as NDF (*p* = 0.02) and ADF (*p* = 0.006) than the diets supplemented with rapeseed and linseed groats. The rapeseed- and linseed groat-supplemented diets did not differ statistically.

#### 3.3.4. Nitrogen

Nitrogen is excreted mainly in urine. It seems that N remained better in the frozen urine with no added acid, shown by higher concentrations (Table 5). Regardless of the storing method (acidified or not), the protein feed supplemented diets increased the urine N concentration in hay-containing diets (*p* = 0.061–*p* < 0.001). The protein feed supplemented diets did not differ statistically in the N concentration of urine.

The N excretion in dung was largest in horses fed the forage-only diets (A and B). Supplementing the hay diet with oats and/or protein feeds decreased N excretion in faeces (*p* = 0.087–0.001). Feeding with the haylage-only diet resulted in statistically significantly larger N excretion in dung than in other diets (*p* = 0.002). However, it was almost equal to the hay-only diet (difference not tested). A comparison between the hay-only diet and the supplemented diets also showed statistically significant difference (*p* = 0.001). When supplemented, the diets with a protein feed, soya-supplemented diet produced less faeces N than the other supplemental feeds (*p* = 0.005).

The total N excretion (dung + urine) was smallest in the hay-only diet; a comparison with the supplemented diets showed a highly statistically significant (*p* < 0.001) difference. The hay + oats diet caused smaller total excretion than the protein feed-supplemented diet, the difference depending on the urine storage method (*p* < 0.001 acidified urine; *p* = 0.034 non-acidified urine).

### 3.4. Blood Parameters

Blood serum urea level of the horses on haylage-only diet tended to be higher (*p* = 0.092) than in the horses on the other diets (Table 7). Erythrocytes were higher in the horses on hay-only diet than in the horses on the supplemented diets (C–F) (*p* = 0.001), and leucocytes were somewhat lower (*p* = 0.033) in the horses fed the hay + oats diet compared to the horses on the protein feed-supplemented diets (D–F).

## 4. Discussion

### 4.1. Feed Composition and Nutrient Intake

The CP content of hay and haylage was higher than the average values reported for Finnish forages produced for horses [4], both feeds being of “medium nutritional quality”. The CP content of oats was lower than that given in the Finnish Feed Tables and Feeding Recommendations (10.7 vs. 12–13%). The NDF and values were also lower than the values presented for medium quality Finnish oats [26].

The OM and ADF content of hay and haylage was close to each other, but haylage had higher NDF than hay. The variation of haylage DM content may be due to moisture evaporation from the big bales after they were open. The feed values of protein feeds were close to the values presented in the Finnish Feed tables and feeding recommendations [26].

The nutrient intakes naturally varied due to the differences in the composition of the diets and actual intakes, although the individual diets were initially formulated and balanced to correspond to the needs of each horse [26] and to be as isocaloric as possible. The mean daily energy intake of 83.3 MJ ME during the experiment was well within the recommendations for horses involved in light work [26]. The average DM intake of 1.3–1.5% of the BW was at the lower limit of the recommendations [26,38]. The smaller DM, CP and ME intake of the horses on the haylage-only diet was due to the smaller DM content of haylage during the first period than in the following periods (59.9 vs. 62.3–65.1%), which is why we excluded the CP intake result of the horse in the first period from the analysis. In addition, the size of the horse affected the individual daily portion, such that larger horses had larger portions.

Because of the low CP content in hay (82 g/kg DM) and haylage (111 g/kg DM), the protein intakes of the forage-only diets were somewhat lower than in the Finnish recommendations [26] but met the NRC-2007 [38] recommendations. The forage samples were analysed prior to the experiment to adjust the diets, but the hay fed varied in its feed values and was not as good as shown by the primary analyses. As Table 1 shows, the diets were not isonitrogenous as was the original aim. However, the differences in CP between the protein feed-supplemented diets were minor, but above the recommendations.

The horses maintained their BW and BCS during the experiment, indicating that the feeds, feeding regime and intakes applied covered the nutritional needs of the horses during the experimental period.

### 4.2. Digestibility and Nutrient Excretion

Improved CP (N) digestibilities with an increasing grain CP content of the diet have been reported in several studies [28,39,40,41,42], which supports our results. According to Trottier at al. [43], differences in N digestibility are also related to the diet’s fibre composition, because lignification decreases cell wall protein digestibility.

There were no differences in the fibre (NDF, ADF) digestibility between the supplemented diets because of the minor differences in fibre intakes. Instead, the forage-only diets had higher fibre component intakes than the other diets, resulting in lower OM, DM and CP digestibilities, and greater excretion of these components and nutrients. The digestibility values observed are comparable with those reported previously for Finnhorses of the same age on forage-rich grain- and linseed groat-supplemented diets [28,40,44].

In studies, lower precaecal N digestibilities have been observed for forages than protein supplements such as soya bean meal [45,46]. In the present study, supplemented oats and protein feeds improved N digestibility. Forages alone did not offer protein that was as well digested, and they had a poorer AA composition than grain- and protein feed-supplemented diets. However, early-cut forage with low fibre content may have high CP digestibility (0.92), and can be used in forage-only diets of high-performance horses [3]. The researchers recommended low-digestibility (0.45–0.50) late-cut forage only for horses on the maintenance level [3]. Formulating diets, it should be considered that there are differences in cell wall digestibility between grasses grown in different geographical areas (climatic zones): at lower temperatures (e.g., in northern latitudes), the digestibility is better than in high-temperature climates [47,48]. For example, in Iceland high CP digestibilities for forages have been reported partly because of low ambient temperatures [3].

### 4.3. Faeces, Urine and N Excretion

Urine and faeces excretions depend on the diet fed, but the amounts measured in this study corresponded to those reported in the literature for horses of the same size; 9–12 L urine and 12–17 kg dung [49,50,51,52]. However, smaller urine excretions have also been reported for adult horses [10]. Forage-only (haylage or hay) diets in this study increased the production of both faeces and urine. In previous studies, the urine amount has been largest in the highest CP intakes [11,51,53]. The urine amounts in the present study have a certain inaccuracy because of the problems with the collecting harnesses described previously. Weir et al. [12] used similar harnesses and also reported some possible losses of N due to evaporation from the harnesses.

Excreted N originates mainly from urine because the main way horses excrete N is through it [52,54]. The amount of N and other nutrients excreted by the horse depends on nutrition and feed quality [42,43]. Large urine excretion results in the large renal excretion, i.e., renal N excretion correlates positively with CP intake [11,51].

Forage-only diets in the present study increased N excretion in dung. The N in the dung of forage-only fed horses corresponded to that reported by Keskinen et al. [16] in horses fed with a 100% forage diet or with very low grain levels. In the study of Graham-Thiers and Bowen [42], faecal N excretion was greater for the hay-only-fed horses than in horses fed hay and grain. In high-fibre feeds, protein may pass enzymatic digestion in the small intestine and go to the lower digestive tract to be fermented by the microbiota producing microbial protein, which is not efficiently absorbed, resulting in higher amounts of N in dung [41,55,56]. In this study, N excretion in dung was largest in horses fed the forage-only diets and, supplementing with oats and/or protein feeds decreased N excretion in faeces. Supplementing hay with oats and protein feeds can be assumed to improve the AA composition of the diets: the AA content differed in the supplemental protein feeds used [26], the best AA composition being in soya groats. To supplement diets having poor AA content (e.g., forage-based diets) with a good AA profile feed is, thus, recommendable, which is supported by literature [27,42]). Hainze et al. [57] reported that an increase of feed CP content significantly increased N excretion in faeces, but according to the study of Potts et al. [58] N amount in forages did not influence the N in faeces.

In this study, soya groat-supplementation produced less faecal N, but not less urinary N, than in the other supplementations. Oliveira et al. [59] found that soya digested well, but extra N was excreted in urine. Many studies reported elevated N lost in urine when the CP content in the feeds and CP intake was increased [41,58,59,60].

Concerning forages, Ragnarson and Lindberg [3,41] reported that N excretion increased when the cutting of the hay is delayed, which may be due to the increment of the cell wall material releasing phenolic acids [11]. According to one study [61], 65% of the total plant AA in timothy is in the NDF fraction. Preserved grass products (e.g., hay, silage, cobs) led to higher urine and N excretion (per g of ingested CP) than grains, protein supplements and sugar beet pulp, for example [11], which supports the results of the present study.

Graham-Thiers and Bowen [42] concluded that the better AA profile and larger intake and better digestibility of AA in grain-supplemented horses resulted in greater N excretion via urine than the horses fed hay only. Because of the problems we faced in collecting urine, we cannot make any final conclusions regarding urine N excretion. However, the N concentrations seemed to remain better in the frozen urine with no added acid. Conflicting with this, Weir et al. [12] suggested that added acid may have resulted to higher urinary N concentrations in the study of Eckert et al. [62], who added acid to urine samples. In the present study, the amount of acid was always constant in acidified urine samples, but the amount of urine stored in the acid varied according to how much urine was recovered from each urination. As a result, the ratio of urine to acid differed in different samples. There were therefore some differences in the combined N concentrations (faeces + urine) due to differences in the preservation method.

AA content and composition plays an important role in N excretion, and the better it corresponds to the animal’s needs, the lower are the protein (N × 6.25) intakes that can be applied [42]. In one study, total N excretion was reduced by 10% in faeces and 45% in urine when dietary CP intake was close to the requirement [12]. It was aimed to feed horses in the present study in accordance with the recommendations of horses involved in light work. However, the protein feed-supplemented diets exceeded the recommendations (by approximately 8–14%), and on the contrary, the forage-only diets and hay + oat diet offered intakes below the recommended intakes. This evidenced how difficult it is to formulate the daily feed portions in practice.

The recommendations do not take the protein source into account. When good-quality protein is fed, smaller N intakes can be applied to enhance the increased N excretion, at least concerning adult horses on a low-intensity level of exercise. This is supported by Trottier et al. [43] who suggest that feeding to meet minimum N requirements is the most effective way of decreasing N excretion and, therefore of minimizing leaching to waters. It is therefore important to consider the protein source and protein quality, aiming to improve protein digestibility and availability, and to decrease N excretion, which is supported by the conclusion of Hainze et al. [57] that feed ingredient selection represented a viable approach to dietary nutrient management for meeting regulations pertaining to environmental disposal of manure from equine operators.

Resulting from the above, in stable and horse management, improved feeding planning and practices are ways of decreasing the risk of N leaching and evaporation. Large variation in feeds concerning the protein quality and digestibility may exist, which is why it is important to ensure the good nutritional value of the feed by harvesting the forage crop at an appropriate growth stage (not too late) to minimize the N loss when forage-only or forage-rich diets are fed. The risk of excess N lost is largest when low-activity (sedentary) horses are fed with large amounts of forage adjusted to their energy needs. Furthermore, from an economic perspective it is irrelevant to feed excess protein, because protein is an expensive component of feeds. According to Virkajärvi et al. [63] knowledge of plant physiology provides tools to understand changes in forage quality. To meet nutritional requirements, different forage batches need to be analysed, requirements defined and correct feeds fed to various horse categories.

In addition, it is important to retain the N in the bedding of stabled horses, recognizing that various bedding materials differ in their N retention capacity [19,20,64,65]. Removing faeces from paddocks is also an efficient practice to minimize the leaching risk [66,67,68]. This is especially recommended to do daily, if the space allowance is less than 200 m^2^ per horse [68]. The CP nutrition and N excretion via urine also influence stable air quality. Urinary N is the main source of NH_3_-volatilised from equine manure [12], and NH_3_ in large amounts may be detrimental to horses’ and people’s health [69].

Although horse manure may have some value as a fertilizer, its N content is generally low for this purpose. In addition, due to net N mobilization, horse manure is not a desirable fertilizer. Nitrogen can be lost if manure is not handled properly, but N leaching from composted manure is smaller than from fresh manure [70]. Maximizing nutrient circulation and utilization is a key factor affecting the environmental impact of horses. Based on the data of this study, the calculatory potential annual N amount produced by a horse (BW 550 kg [25]), and which can be used in farming, is 42.3–67.9 kg. This accounts for 22–35% of the N fertilization needs of horse pastures (in Finland [71]). However, the proportion of water-soluble N available to plants is only around 30 to 40%, depending on the bedding material [16].

Nitrogen leaching into waters is not the only environmental risk, but gas emissions may also occur. N evaporates in the form of NH_3_, N_2_ and NO gases [18,19]. Ammonia volatilization is generally the major pathway for N losses from manure [20,21]. According to Maljanen et al. [19], the evaporation increases during manure storage because of the mineralization/nitrification of N. They found that emissions from horse dung were larger than the mean emissions observed from dairy cow dung in their earlier studies. However, leaching and emissions depend on the bedding material [16,18,65].

We did not report and analyse the N balances because of the problems in urine collection. We assumed that the urine excretion was overestimated, explaining the fact that the balances seemed to result in negative values, as can be concluded based on the differences between N intake (Table 3) and excretion (Table 5) values.

### 4.4. Blood Parameters

Erythrocytes were higher in the horses on the hay-only diet than in the horses on the supplemented diets, and leucocytes somewhat lower in the horses received the hay + oats diet, compared to the leucocyte levels of horses on the protein-supplemented diets. However, both the erythrocyte and leucocyte values were within the normal ranges given for Finnhorses [37]. Further, the horses showed no signs of anaemia or inflammations, or any diseases. No reasons for the elevated values can therefore be given.

Horses on the haylage-only diet tended to have higher blood urea values, but we did not calculate the correlation between urea content and N excretion because of the urine collection problems mentioned previously. However, in the literature, a strong positive linear relationship between blood urea concentration and the rate of excretion has been reported in horses, meaning that blood urea can be used to quantify N utilization and excretion rates [72]. Kohn et al. [72] found that N excretion increased when blood urea was high. In addition, Graham-Thiers and Bowen [42] found that excess AA and CP intakes increased the serum urea concentration. In the present study, CP digestibility in the haylage-only diet was lower and faeces N excretion higher than in the protein feed-supplemented diets. The CP intake of the horses was approximately at the same level with the protein feed-supplemented diets.

## 5. Conclusions

Horses excreted more nitrogen in their urine than in dung. The results showed that feed choices in the feeding of horses affected the amount of nitrogen excreted by horses. Horses excreted the least nitrogen when fed dry hay only compared to the hay + oats diet supplemented with feeds rich in protein. Feeding recommendations should consider not only the horse category and work level, but also the protein source. When feeds with good protein quality and AA profile are fed, smaller N intakes can be applied to minimize the risk of the N excretion, at least concerning adult horses involved in light exercise. At the farm level, improved understanding of feed quality, feeding planning and practices, as well as binding the excreted N in the beddings, is a way to decrease the risk of N leaching and evaporation. Further research on feeding strategies, their applications and selection of protein supplements for horses is essential to reduce the horse industry’s harmful impacts on the environment.

## Figures and Tables

**Table 1:** Average formulation of the experimental diets (as fed kg/d).

Diet/Feed	Haylage	Hay	Oats	Soya Groats	Rapeseed Goats	Linseed Groats
A	11.7	-	-	-	-	-
B	-	9.5	-	-	-	-
C	-	6.6	2.8	-	-	-
D	-	6.6	2.4	0.4	-	-
E	-	6.7	2.1	-	0.8	-
F	-	6.6	2.0	-	-	0.8

**Table 2:** Average chemical composition (g/kg DM) and energy value (ME MJ/kg DM) of the experimental feeds (g/kg DM).

Feed/ Nutrient	Haylage	Hay	Oats	Soya Groats	Rapeseed Groats	Linseed Groats
DM	627	849	856	897	880	926
OM	938	940	966	930	928	939
CP	111	83,2	107	495	364	301
NDF	617	604	288	114	280	264
ADF	329	327	123	57,7	185	142
Ash	61.9	60.5	33.8	70.3	72.5	60.7
ME MJ	9.7	9.7	12.3	13.4	11.9	15.9

DM = dry matter; OM = organic matter; CP = crude protein; NDF = neutral detergent fibre; ADF = acid detergent fibre; ME MJ = metabolizable energy in megajoules.

**Table 3:** Daily nutrient (kg) and energy (MJ ME) intakes and statistical differences between the experimental diets.

Statistical Significancy (*p*-Values)												
	A Haylage	B Hay	C Hay	D Hay	E Hay	F Hay	Diet	A	B	C	D	E
Supplemental feed/Nutrient	-	-	Oats	Oats + Soya	Oats + Rapeseed	Oats + Linseed	SEM	vs. B and C–F	vs. C–F	vs. D–F	vs. E, F	vs. F
DM	7.52	8.09	8.06	8.13	8.04	8.23	0.167	<0.001	0.790	0.446	0.962	0.106
OM	6.96	7.55	7.54	7.60	7.49	7.69	0.160	<0.001	0.694	0.561	0.900	0.094
CP	0.85	0.67	0.72	0.87	0.89	0.88	0.015	<0.001	<0.001	<0.001	0.097	0.026
dCP	0.48	0.42	0.45	0.55	0.57	0.58	0.031					
N	0.14	0.11	0.12	0.14	0.14	0.14	0.003	0.167	<0.001	<0.001	0.556	0.394
NDF	4.57	4.82	4.05	4.03	4.03	4.12	0.096	<0.001	<0.001	0.864	0.490	0.281
ADF	2.44	2.61	2.11	2.11	2.15	2.17	0.048	<0.001	<0.001	0.336	0.181	0.586
Ash	0.46	0.48	0.42	0.44	0.45	0.45	0.009	0.145	<0.001	0.005	0.118	0.620
ME MJ	76.4	79.1	84.0	85.2	85.9	97.4	0.43	-	-	-	-	-

DM = dry matter; OM = organic matter; CP = crude protein; dCP = digestible crude protein; N = nitrogen; NDF = neutral detergent fibre; ADF = acid detergent fibre; ME MJ = metabolizable energy in megajoule; n = 24, except n = 23 for CP.

**Table 4:** Nutrient digestibilities of the experimental diets.

Statistical Significancy (*p*-Values)												
Diet Forage type	A Haylage	B Hay	C Hay	D Hay	E Hay	F Hay	Diet	A	B	C	D	E
Supplemental feed/Nutrient	-	-	Oats	Oats + Soya	Oats + Rapeseed	Oats + Linseed	SEM	vs. B and C–F	vs. C–F	vs. D–F	vs. E, F	vs. F
DM	0.515	0.560	0.604	0.624	0.598	0.599	0.0097	<0.001	<0.001	0.722	0.019	0.909
OM	0.528	0.573	0.620	0.643	0.615	0.614	0.0088	<0.001	<0.001	0.597	0.006	0.918
CP	0.609	0.514	0.632	0.703	0.671	0.663	0.0193	0.034	<0.001	0.002	0.016	0.588
N	0.609	0.514	0.632	0.703	0.671	0.663	0.0193	0.034	<0.001	0.002	0.016	0.588
NDF	0.484	0.507	0.478	0.500	0.473	0.478	0.0124	0.773	0.051	0.595	0.063	0.697
ADF	0.474	0.499	0.452	0.485	0.463	0.460	0.0134	0.866	0.008	0.148	0.059	0.830
Ash	0.212	0.215	0.251	0.290	0.225	0.248	0.0408	0.409	0.357	0.722	0.019	0.909

DM = dry matter; OM = organic matter; CP = crude protein; N = nitrogen; NDF = neutral detergent fibre; ADF = acid detergent fibre; n = 24, except n = 23 for ash.

**Table 5:** Excretion of dung, urine and nitrogen (N) per day (d).

Statistical Significancy (*p*-Values)												
Diet Forage type	A Haylage	B Hay	C Hay	D Hay	E Hay	F Hay	Diet	A	B	C	D	E
Supplemental feed	-	-	Oats	Oats + Soya	Oats + Rapeseed	Oats + Linseed	SEM	vs. B, C–F	vs. C–F	vs. D–F	vs. E, F	vs. F
Dung (fresh) kg	19.3	18.7	14.4	14.1	13.9	14.0	0.88	<0.001	<0.001	0.512	0.744	0.831
Dung DM kg	3.6	3.6	3.2	3.1	3.2	3.3	0.06	<0.001	<0.001	0.738	0.012	0.613
Urine L	14.0	14.3	10.9	13.0	12.0	11.8	0.86	0.026	0.003	0.050	0.146	0.827
N in dung g	52	51	43	42	47	48	2.27	0.002	0.001	0.087	0.005	0.603
N in urine g (unacidified)	123	85	117	131	139	124	7.09	0.644	<0.001	0.061	0.950	0.106
Urine N g (acidified)	104	64	81	111	111	105	5.79	0.144	<0.001	0.001	0.678	0.464
N dung + urine g (unacidified)	175	136	160	173	186	172	7.96	0.239	<0.001	0.034	0.464	0.118
N dung + urine g (acidified)	156	115	124	153	158	153	5.41	0.016	<0.001	<0.001	0.644	0.427

**Table 6:** Nutrient excretion (kg) in dung per day (d).

Statistical Significancy (*p*-Values)												
Diet Forage type	A Haylage	B Hay	C Hay	D Hay	E Hay	F Hay		A	B	C	D	E
Supplemental feed	-	-	Oats	Oats + Soya	Oats + Rapeseed	Oats + Linseed	SEM	vs. B, C–F	vs. C–F	vs. D–F	vs. E, F	vs. F
DM	3.63	3.57	3.17	3.05	3.25	3.29	0.058	<0.001	<0.001	0.738	0.012	0.613
OM	3.27	3.23	2.85	2.71	2.90	2.96	0.050	<0.001	<0.001	0.976	0.004	0.393
CP	0.32	0.32	0.26	0.26	0.29	0.30	0.014	0.002	<0.001	0.077	0.007	0.683
NDF	2.35	2.38	2.10	2.01	2.14	2.14	0.041	0.001	<0.001	0.963	0.020	0.969
ADF	1.28	1.30	1.15	1.08	1.16	1.17	0.022	0.001	<0.001	0.534	0.006	0.645
Ash	0.36	0.34	0.32	0.32	0.35	0.33	0.012	0.049	0.440	0.567	0.224	0.431

**Table 7:** Blood serum parameters of the horses on the experimental diets. Reference values [37] for Finnhorses are presented in brackets.

Statistical Significancy (*p*-Values)												
Diet Forage type	A Haylage	B Hay	C Hay	D Hay	E Hay	F Hay	Diet	A	B	C	D	E
Supplemental feed	-	-	Oats	Oats + Soya	Oats + Rapeseed	Oats + Linseed	SEM	vs. B, C–F	vs. C–F	vs. D–F	vs. E, F	vs. F
Leucocytes × 10⁹/L (4.8–9.5)	6.5	6.3	5.5	6.4	6.0	6.4	0.40	0.188	0.398	0.033	0.481	0.375
Erythrocytes × 10¹²/L (6.6–9.7)	6.7	7.4	6.7	6.3	6.8	6.6	0.27	0.794	0.001	0.450	0.101	0.363
Haemoglobin g/L (118–159)	115	120	116	112	116	114	3,64	0.714	0.024	0.449	0.203	0.354
Haematocrit % (34–43)	33.9	35.4	34.3	33.2	34.3	33.3	1.10	0.790	0.033	0.347	0.441	0.252
Serum urea mmol/L (3.3–7.8)	5.0	4.0	4.2	4.4	5.1	4.3	0.34	0.092	0.157	0.343	0.465	0.100
Serum protein g/L (55–75)	61.5	62.6	62.2	62.0	61.6	61.8	1.24	0.575	0.466	0.692	0.763	0.935

## Data Availability

The data are not publicly available due to the authors’ further use of the data for academic work.
