# Correction: Saastamoinen et al. Protein Source and Intake Effects on Diet Digestibility and N Excretion in Horses—A Risk of Environmental N Load of Horses. *Animals* 2021, *11,* 3568

**DOI:** 10.3390/ani12070848

**Published:** 2022-03-28

**Authors:** Markku Saastamoinen, Susanna Särkijärvi, Heli Suomala

**Affiliations:** 1 Production Systems, Natural Resources Institute Finland (Luke), 31600 Jokioinen, Finland; susanna.sarkijarvi@roviopetfoods.fi; 2 Department of Animal Science, University of Helsinki, 00790 Helsinki, Finland; heli.suomala@hevostietokeskus.fi

## Text Correction

There was an error in the original publication [1]: there is a typing error in the Materials and Methods Section. In the text, it was incorrectly said that the method was based on urine creatine concentration. However, correctly, the method is based on creatinine concentration.

A correction has been made to **2.4. Faeces and Urine Sampling and Analyses**:

On some days, the collection of all the urine was unsuccessful, with losses due to difficulties in keeping and fitting the harnesses tightly on the mares. The daily amount of urine was therefore calculated based on its creatinine concentration in the urine [30]: Y = 24.3 + (14067/x), where y is the urine mount, and x is the creatinine concentration in the urine (creatinine molecular weight 0.13113 g/mmol). The N balances were not calculated because of the uncertain urine collection method and calculated values.

The faeces and urine samples were analysed at Luke Laboratories. Faeces samples were analysed using the same methods for dry matter (DM), crude protein (CP), NDF, and ADF and ash as feed samples, as described above. The faeces were dried in an oven for 20 h (2 h at + 60 °C, 18 h at +110 °C) and milled (1 mm sieve) for the analyses. The fresh sample was also analysed for N content using the Khjeldal method. Urine samples were analysed for nitrogen (Khjeldal method) and creatinine concentrations for both the pure and acid-treated urine. The analysis of creatinine was based on the photometric method of Jaffe (see, e.g., [31]), using a wavelength of 510 nm. The mean urinary specific gravity of the horses reported in the literature was 1.034 g/L [32–36].

The authors apologise for any inconvenience caused and state that the scientific conclusions are unaffected. The original publication has also been updated.
